# On the Social-Relational Moral Standing of AI: An Empirical Study Using AI-Generated Art

**DOI:** 10.3389/frobt.2021.719944

**Published:** 2021-08-05

**Authors:** Gabriel Lima, Assem Zhunis, Lev Manovich, Meeyoung Cha

**Affiliations:** ^1^ School of Computing, Korea Advanced Institute of Science and Technology, Daejeon, Korea; ^2^ Data Science Group, Institute for Basic Science, Daejeon, Korea; ^3^ The Graduate Center, City University of New York, New York, NY, United States

**Keywords:** artificial intelligence, moral standing, moral status, agency, experience, patiency, art, rights

## Abstract

The moral standing of robots and artificial intelligence (AI) systems has become a widely debated topic by normative research. This discussion, however, has primarily focused on those systems developed for social functions, e.g., social robots. Given the increasing interdependence of society with nonsocial machines, examining how existing normative claims could be extended to specific disrupted sectors, such as the art industry, has become imperative. Inspired by the proposals to ground machines’ moral status on social relations advanced by Gunkel and Coeckelbergh, this research presents online experiments (*∑N = 448*) that test whether and how interacting with AI-generated art affects the perceived moral standing of its creator, i.e., the AI-generative system. Our results indicate that assessing an AI system’s lack of mind could influence how people subsequently evaluate AI-generated art. We also find that the overvaluation of AI-generated images could negatively affect their creator’s perceived agency. Our experiments, however, did not suggest that interacting with AI-generated art has any significant effect on the perceived moral standing of the machine. These findings reveal that social-relational approaches to AI rights could be intertwined with property-based theses of moral standing. We shed light on how empirical studies can contribute to the AI and robot rights debate by revealing the public perception of this issue.

## 1 Introduction

As robots and artificial intelligence (AI) systems become widespread, scholars have questioned whether society should have any responsibility towards them. This inquiry, also called the “robot rights” debate (Gunkel, 2018b), comprehensively questions whether these systems matter morally, i.e., whether a certain level of moral standing should be granted or recognized to them. Scholars have expressed a plurality of views on this topic. Those who oppose the prospect denounce the idea by arguing that these entities are ontologically different from humans (Miller, 2015). Others argue that this debate occurs at the expense of more salient moral issues (Birhane and van Dijk, 2020) and could lead to social disruption (Bryson, 2018). In contrast, some scholars propose that robots and AI systems should matter morally if they develop consciousness or sentience (Torrance, 2008). Even if they don’t become conscious, society might choose to protect AI and robots to discourage immoral human behavior (Darling, 2016).

This research is motivated by the proposals advanced by Gunkel and Coeckelbergh, both of whom advocate a social-relational perspective to the robot rights debate. Gunkel (2018a) proposes that moral status is grounded on social relations rather than an entity’s ontology, such that automated systems could matter morally in the face of social interactions. In a similar vein, Coeckelbergh (2020b) argues that society could give these entities moral standing due to their extrinsic value to humans and suggests that these entities could be granted indirect moral status according to how much humans value them.

The AI and robot rights discussion has been mostly restricted to normative research. Few empirical studies have examined the public attitude towards these systems’ moral standing (Lima et al., 2020; de Graaf et al., 2021). These studies have also not addressed specific perspectives advanced by previous normative work. This paper thus investigates whether social-relational approaches to this debate could be extended to a significant nonsocial robotics context, namely AI-generated art. AI-generative systems have achieved impressive results in generating a wide range of image styles (Karras et al., 2019; Goodfellow et al., 2014). Some of these images have been auctioned in the real world for remarkable prices (Cohn, 2018; Ives, 2021). Considering the social dimension of art, we inquire whether interacting with AI-generated art influences the perceived moral status of its creator, i.e., the AI-generative system.

After carefully selecting a series of AI-generated paintings (Experimental Setting, *N* = 45; Section 4), we conducted two studies inspired by the social-relational approaches advanced by Gunkel (Study 1, *N* = 140; Section 5) and Coeckelbergh (Study 2, *N* = 263; Section 6). Study 1 inquired whether interacting with AI-generated art modifies how participants perceive an AI systems’ agency and patiency through a mind perception questionnaire (Gray et al., 2007). Study 2 examined whether highlighting an AI system’s extrinsic value by undervaluing or overvaluing its outputs affects its perceived agency, patiency, and moral status.

Both studies show that participants deemed AI-generative systems as able to create and experience art to a significant level. Study 1 identified that nudging participants to think about an AI system’s “mind” negatively influenced how they judged its artwork; this indicates that ontological considerations could play a role in interactions with non-human entities. Moreover, Study 2 found that people shown overvalued AI-generated images may undermine its creator’s agency compared to other control conditions. However, none of the studies suggested that interacting with AI-generated art would influence people’s perception of the AI system’s moral standing. Collectively, our results reveal that considerations about the mind of non-humans could be intertwined with social-relational theses of their moral standing.

We discuss how studies like ours can contribute to the robot rights debate by obtaining empirical data supporting or challenging existing normative proposals. Scholars posit that public perceptions of AI systems could partially shape their development, use, and regulation (Cave and Dihal, 2019). Studies such as ours can thus inform future discussions on how the general public perceives AI’s and robots’ moral and social standing. We also propose future research directions, such as understanding how ontological considerations could play a role in human-robot interactions and whether our results extend to other environments where AI and robots are deployed.

## 2 Background

### 2.1 Moral Status of Artificial Intelligence and Robots

Extensive literature has questioned who should be responsible for the actions of artificial intelligence (AI) and robotic systems. Some scholars propose the existence of a responsibility gap, where no entity can be appropriately held responsible for harms caused by these entities (Matthias, 2004; Asaro, 2016). Others argue that worries about a responsibility gap are overstated (Tigard, 2020) and designers should instead proactively take responsibility for their creations (Johnson, 2015). The discussion surrounding the responsibility gap (or its nonexistence) questions AI systems’ moral agency, i.e., their capacity to do right or wrong. In this research, we instead follow the perspective that asks whether these systems can be subjects of rights and wrongs, i.e., whether they can (and should) be moral patients (Gunkel, 2012).

While a moral agent can act morally and possibly be deemed responsible for its actions, to be a moral patient implies that society has responsibilities towards it (Bryson, 2018). Moral patients have a certain moral status, hence suggesting that they have legitimate interests that other agents should consider, i.e., there are constraints on how one treats a moral patient (Gordon, 2020). Extensive philosophical literature has debated which conditions ground moral status. A common perspective is that moral patiency (and agency) depends on an entity satisfying specific properties (Coeckelbergh, 2014). Some scholars argue that sentience and consciousness are necessary conditions for moral patiency (Bernstein, 1998). Nevertheless, these views are rarely agreed upon, particularly in the literature discussing the moral status of non-humans (Gellers, 2020).

The debate around the moral patiency of AI systems and robots has often been framed under the umbrella of “robot rights” (Gunkel, 2018b). This setting relies on the fact that high moral status (e.g., moral patiency) grounds moral personhood, which in turn ascribes or recognizes an entity’s moral rights (Gordon, 2020). The robot rights literature challenges the institutions that sort entities by type (e.g., humans, non-human animals, artifacts) and put humans on top. Scholars have argued that reinterpreting the distinction between “who” and “what” may encourage a more respectful, participatory, and dignified social order (Estrada, 2020).

Although the debate’s title might suggest that scholars only propose moral status for embodied systems, research indicates that both robots and (nonphysical) AI systems could have their moral patiency recognized (e.g., see Bryson (2018); Lima et al. (2020)). Throughout this paper, we refer to “robot rights” for consistency with previous work on the topic but do not necessarily restrict our discussion to embodied systems. The series of studies covered by this research specifically address systems without any physical presence in the world, i.e., AI-generative models, and we often use “AI” and “robots” as synonyms.

Some scholars opposed to robot rights argue that its mere conception is unthinkable and should be denounced. For instance, Birhane and van Dijk (2020) argue that this debate occurs at the expense of more urgent ethical issues, such as privacy and fairness, and should be avoided at all costs. That is not to say that all scholars who oppose robots and AI systems with any moral status discard its possibility. Bryson (2018), for instance, recognizes that such systems could be accorded rights but opposes it. Bryson argues that creating systems that could be granted certain moral status is bound to conflict with a coherent ethical system and thus should be avoided.

Another series of arguments against recognizing automated agents’ moral status relies on their incompatibilities with what authors defend to be moral patiency preconditions. Miller (2015) has argued against robot rights under the justification that robots are ontologically different from humans. Being created for a specific purpose, robots are not brought into the world similarly to humans. Miller defends that humans’ lack of purpose lays the foundation of their rights, as they allow humans to discover their purpose. While this argument defends that granting robots and AI systems certain moral status should be denounced regardless of whether they satisfy specific properties, other scholars are disposed to granting or recognizing robots’ and AI’s rights if (and only if) they develop them. Torrance (2008) is one author that is open to granting moral status to automated agents if they become conscious or sentient. A distinct approach has been put forth by Danaher (2020), who proposes to use behavioral inferences as evidence of the ontological attributes that ground moral status. Such proposal posits that automated agents could be granted significant moral status if they behave similarly enough to entities with high moral status.

Various authors’ perspectives to the discussion of AI and robot rights propose to ground these systems’ moral patiency not on themselves but on those who interact with them. This indirect approach often suggests protecting automated agents for the sake of humans. For instance, Darling (2016) defends that society should protect social robots from cruelty to not promote such immoral behavior in human-human interactions. In a similar vein, Nyholm (2020) argues that we should respect anthropomorphized robots’ apparent humanity out of respect for human beings’ humanity. Friedman (2020) reinterprets the standard dyadic conception of morality and defends the protection of perceived robotic moral patients by viewing humans as both moral agents and patients of their actions towards robots. A similar approach has also been put forward by Coeckelbergh (2020a), who argues that engaging in immoral behavior towards social robots could damage an agent’s moral character (i.e., its virtue), and thus should be avoided.

The present research builds upon the social-relational perspectives to robot rights put forth by Gunkel and Coeckelbergh. Inspired by the relational turn in ethics concerning non-human animals (Taylor, 2017), humans (Levinas, 1979), and the environment (Naess, 2017), both authors argue against property-based conceptions of moral patiency and defend instead that social relations ground moral status. Gunkel (2018b) argues for a direct approach to robot rights such that moral status is grounded on one’s response to a social encounter with a robotic other. The author defends that moral persons are not defined by their ontological attributes but by how they engage in social relations. As Gunkel (2012) himself puts it, “moral consideration is decided and conferred not based on some pre-determined ontological criteria […] but in the face of actual social relationships and interactions.”

Coeckelbergh’s perspective differs from Gunkel’s in that it gives indirect moral standing to robots or AI systems “based on the ways humans […] relate to them” (Coeckelbergh, 2020b). Although also relying on how humans interact with automated agents, his argument posits that their moral standing should instead be grounded on their extrinsic value to humans (Coeckelbergh, 2010). If humans, who are valuable per se, value robots and AI systems, the latter could also be deemed morally valuable based not on themselves but on the entity who ascribes their value. We return to these social-relational approaches to AI and robot rights in Section 3 when motivating our series of empirical studies on people’s perception of AI systems’ moral standing.

### 2.2 Mind Perception Theory

The conceptions of moral patiency (and agency) presented above rely on philosophical interpretations of robots’ and AI systems’ moral standing. A different perspective has been put forward by moral psychology research, which often questions how people perceive entities’ moral status under the Mind Perception Theory. Extensive research (as reviewed by (Gray et al., 2012)) has underscored the importance of people’s ascription of mental capacities in moral judgments and how it maps onto attributions of moral agency and patiency.

A widely used conception of mind perception is that people perceive agents’ and patients’ minds in two distinct dimensions (Gray et al., 2007). The first dimension accounts for entities’ capacities to feel fear, pain, be conscious, and experience other related abilities. Entities perceived to have high levels of this dimension of mind are deemed to have high *experience*, which studies suggest to correlate with the conferring of moral rights (Waytz et al., 2010). The second dimension of mind perception—termed *agency*—includes the capacity of self-control, morality, planning, thought, and others notions related to an entity’s moral agency. Previous research has observed perceived agency to be linked to attributions of responsibility Gray et al. (2007).

Mind perception in the context of robots and AI systems has received significant attention in previous work. Gray et al. (2007) have found robots being ascribed moderate levels of agency and low levels of experience. In the context of economic games, Lee et al. (2021) have observed electronic agents being ascribed moral standing if systems were manipulated to possess high agency and patiency traits. Previous work has also found systems expressing emotions (e.g., with high experience) being offered larger amounts of money in economic exchanges than their low-experience counterparts (de Melo et al., 2014). In summary, previous research broadly suggests that people’s ascription of agency and experience to automated agents plays a role in their interaction with these systems. Building upon the aforementioned social-relational approach to electronic agents’ moral standing, we instead inquire whether interacting with these systems influences perceptions of their patiency (and agency), i.e., how people perceive their mind and corresponding moral status.

### 2.3 Artificial Intelligence-Generative Models

Much of the work on robots’ moral status covers those systems developed for social functions, e.g., social robots. Nevertheless, we note that many of these systems, embodied or not, are not necessarily developed with sociality in mind. Robots and AI systems are currently deployed in various environments, ranging from industrial hangars to decision-making scenarios (e.g., loan and bail decisions). In this study, we distinctively investigate the social-relational approach to electronic agents’ moral standing in the context of AI-generative models.

Extensive research in computer science has been devoted to developing AI-generative models. A wide range of systems have achieved impressive results in the generation of images (Goodfellow et al., 2014; Ramesh et al., 2021), text (Brown et al., 2020), music (Dhariwal et al., 2020), and even patents (Porter, 2020). AI-generated images have received considerable attention by the field, and philosophers have even questioned whether they could be considered art and have defended an open perspective to the possibility of “machine creativity” (Coeckelbergh, 2017).

The deployment of AI-generative systems has raised many ethical and legal questions. Concerned with the environmental and social costs of text-generation models, Bender et al. (2021) have urged researchers to consider the negative societal effects of large language models. AI-generative systems have also posed questions as to who should hold the copyright, intellectual property rights, and authorship of their outputs. Eshraghian (2020) has discussed how “artificial creativity” results from many actors’ efforts and thus poses critical challenges to copyright law. Abbott (2020) has defended that AI systems should be considered authors of their creations so that their creativity can be legally protected. Turner (2018) has gone even further and discussed how AI systems themselves might hold the copyright of their outputs.

Image generation by AI systems has also received considerable attention from the general public. A portrait generated by an AI-generative model was sold for over $430,000 in 2018 (Cohn, 2018), raising questions about the value of “machine creativity.” More recently, a self-portrait of Sophia, the robot which has been granted honorary citizenship in Saudi Arabia, was sold for nearly $700,000 under the premise of it being the first human-robot collaborative art to be auctioned (Ives, 2021). Previous research has also questioned how people perceive art-generated art. Epstein et al. (2020) have shown how people might attribute responsibility for creating realistic paintings to the AI system that generated it, particularly if it is described in an anthropomorphized manner. In a similar vein, Lima et al. (2020) found online users to only marginally denounce the idea of an AI system holding the copyright of its own generated art. Other studies found AI-generated art being evaluated unfavorably vis-a-vis their human-created counterparts (Hong and Curran, 2019; Ragot et al., 2020), even though people do not seem to be able to differentiate between them (Köbis and Mossink, 2021; Gangadharbatla, 2021).

### 2.4 Art as a Social Practice

The present study expands on the social-relational approaches to AI systems’ moral standing in a distinctive environment that was yet to be explored by the literature: AI-generated art. While art is not social in the same way as the social robotics perspective commonly studied by scholars discussing robot rights, art production and evaluation have been often understood as a social process where many entities come together to create what one would call art.

Sociologists of culture have developed a social understanding of the arts under which the artistic production and assignment of value are viewed as social processes involving assistants, curators, galleries, museums, art critics, and many others. The artist is viewed as only one participant of this social undertaking. Many art historians and other humanities scholars also focus on the social aspects of art by showing how artistic canons evolved (i.e., what artists were recognized as “great” was changing), and how many marginalized artists (e.g., women and people of color) were excluded from the history of art (Nochlin, 1971).

One important concept developed first in sociology that later became the common-sense view of art professionals is the “art world.” The art world includes everyone who participates in creating, funding, promoting, exhibiting, writing about, buying, and selling visual art. Art worlds are numerous and extensive by comprising different networks of people. What counts as “art” in each world can also be different. As discussed by Becker (2008), an art world is “the network of people whose cooperative activity, organized via their joint knowledge of conventional means of doing things, produces the kind of artworks that the art world is noted for.” Both the actual objects of art and their meanings result from collective activities, shared understandings, and accepted conventions and norms.

People’s perception of and interaction with art can thus be viewed as a social phenomenon. Rather than seeing our reactions to art as being completely individual and unique, we may assume that they are in part collective—e.g., people with similar backgrounds living in a particular period may have similar tastes. The influential theory in sociology of culture developed by Bourdieu (1984) indeed proposes that people’s taste in the arts is related to their socioeconomic status.

This social paradigm of the arts posits that those who create, evaluate, buy, sell, and interact with art are intertwined in understanding what art is in each art world. The inclusion of AI systems into this environment raises the question of how objects of art and their meaning might be altered in the face of AI-generated art. This revolution might change what society views as art and who people regard as artists that should be included in this artistic social network. We approach this question similar to those who discuss the moral standing of AI systems. Alongside questioning who should be included in the circle of moral patients, we inquire how people embrace AI-generative systems in their art world. We thus question whether interacting with “art” generated by AI systems can influence people’s attribution of moral and artistic status to generative systems.

## 3 Social-Relational Ethics for Robots and Artificial Intelligence

Research on the mind perception of AI has centered on how people’s preconceptions of these systems’ agency and patiency influence future human-machine interactions (e.g., see Lee et al. (2021); de Melo et al. (2014)). However, the social-relational approach to “robot rights” inverts this relationship and instead argues that interacting with automated agents affects how people perceive their moral status. For instance, Gunkel’s proposal of social-relational ethics for grounding the moral status of robots views moral patiency as a result of social interactions, under which people are “obliged to respond [to entities] even before we know anything at all about them and their inner working” (Gunkel, 2018b). Gunkel asserts that moral status does not depend on what the other is or how it came to be but instead emerges from how we respond to “the face of the other” (Gunkel, 2018a).

Gunkel (2018b) discusses how one may anticipate an anthropocentric perspective of an entity’s face by turning it “into a kind of ontological property.” Instead, the author interprets this face to include other entities, such as animals, the environment, technologies, and surely robots. In this work, we expand on this idea and inquire how people respond to the “face” of an AI-generative model. These systems do not have what one would call a face one can respond to but rather output creations that can be interacted with. Study 1 covered by this research questions whether people interacting (i.e., responding) to AI-generative art (i.e., the model’s “face”) influences how they ascribe moral status to its creator.

Coeckelbergh (2010) similarly states that “moral significance resides neither in the object nor in the subject, but the relation between the two,” suggesting that moral status can only be grounded in dynamic social relations. The author highlights that studying robots’ moral considerations must account for how they are deployed and how people might interact with them. In contrast to Gunkel’s, Coeckelbergh’s view on how social-relational ethics can ground robots’ moral status does not rely on how one might respond to electronic agents per se. It instead focuses on how others might value and interact with them, i.e., their extrinsic value.

Coeckelbergh (2020b) has proposed a set of conditions that could sufficiently ground a certain level of indirect moral standing to social robots. These conditions cover how immoral interactions with social robots could denigrate one’s virtue (see also Coeckelbergh (2020a)) and how they could conflict with human-robot relationships. The present research adapts one of these conditions to a nonsocial robot environment. Coeckelbergh proposes that social robots could be given moral standing “if the human user has a (one-directional) relationship to the robot and has developed feelings of attachment and empathy towards the robot” (Coeckelbergh, 2020b). We expand on this view and inquire whether others’ under- or overvaluation of an AI-generative model’s outputs, i.e., whether human users have developed feelings of value towards an AI system, could ground this system’s perceived moral status in Study 2.

It should be noted that the present research’s case study broadens the usual setting discussed by much of the literature on automated agents’ moral standing. Coeckelbergh (2020b) and Darling (2016), for instance, develop their arguments in the context of robots intentionally designed to be integrated into human social environments, i.e., social robots. As mentioned above, however, AI systems are not only deployed in social settings, and scholars have questioned whether they should be granted moral standing in diverse environments (Bryson, 2018; Turner, 2018; Lima et al., 2020). We approach this inquiry through the lens of social interpretations of art, under which artists, curators, galleries, and even laypeople contribute to creating a shared understanding of art, i.e., an art world. This research does not aim to debunk or confirm any of the social-relational approaches to robot rights; it instead seeks to provide a distinct and empirical perspective to the debate.

We present two studies aimed at understanding how social-relational approaches to robots’ moral standing pertain to the context of AI-generative art. We first carefully selected a series of AI-generated images (similar to paintings produced in modern art) that online users could not discern as either human-created or AI-generated. These paintings were used in subsequent studies, and we make them available for future research. Study 1 was influenced by Gunkel’s approach to “robot rights” and evaluated whether interacting with AI-generated images affects how people ascribe patiency and agency to their creator. Finally, Study 2 addressed Coeckelbergh’s proposal of electronic agents’ indirect moral standing by examining whether others’ under- or overvaluation of AI-generated art influences an AI system’s perceived moral status. All studies had been approved by the Institutional Review Board (IRB) of the first author’s institution.

## 4 Experimental Setting

Our experiments presented a series of AI-generated art-looking images to participants and explored whether interacting with AI systems’ outputs influences subsequent ascription of moral status. For that, we employed a state-of-the-art model named StyleGAN2 (Karras et al., 2019) to generate images. StyleGAN2 is based on the Generative Adversarial Network (GAN) architecture (Goodfellow et al., 2014), which consists of two distinct deep neural networks, a generator and a discriminator, that compete with each other during the training process. The generator learns to output data that looks similar to the training set and aims to deceive the discriminator. In contrast, the discriminator tries to distinguish between outputs by the model and the training set’s data. This model architecture has achieved impressive results in a wide range of tasks, ranging from the generation of paintings (Karras et al., 2019) and faces (Karras et al., 2017) to style transfer between images (Zhu et al., 2017).

We generated images using a pre-trained StyleGAN2 implementation available on Github (Baylies, 2020). This model had been trained on a subset of the WikiArt dataset containing over 81,000 paintings. After obtaining an initial set of 200 images, one of the authors with extensive art training selected a subset of 58 images based on their authenticity and quality. We then presented the generated images to participants, who were asked to distinguish which images were generated by AI.

### 4.1 Methods

After agreeing to the research terms, study participants were told that they would be presented with a series of images generated by AI systems and human artists. Note that all images had been generated by the AI-generative model described above. Participants were instructed to indicate who they thought created each image—an AI program or a human artist. Participants were successively shown a random subset of 20 images in random order. Participants also had the option to indicate that they were unsure about its creator for each image. After evaluating all 20 images, participants were debriefed that an AI model had generated all images.

### 4.2 Participants

We recruited 45 respondents (22 men, 21 women, two others; 26 younger than 35 years old) through the Prolific crowdsourcing platform (https://www.prolific.co/; Palan and Schitter (2018)). Participants were required to have completed a minimum of 50 tasks in Prolific with at least a 95% approval rate. All respondents were United States nationals and were compensated $0.87 for the study.

### 4.3 Results

We chose images that were considered most ambiguous based on participants’ ratings. This decision was made by the fact that GAN-based models are intentionally modeled to deceive a discriminator. These models’ training process aims to teach a generator how to output ambiguous images that one cannot discriminate as either real (i.e., human-created) or artificial (i.e., AI-generated). Although another option would be to choose images that participants thought were human-created, we note that doing so could have made future participants suspect the images’ origin. Hence, to mitigate possible deception effects, we decided to discard images that were perceived to have been created by human artists.

**FIGURE 1:**
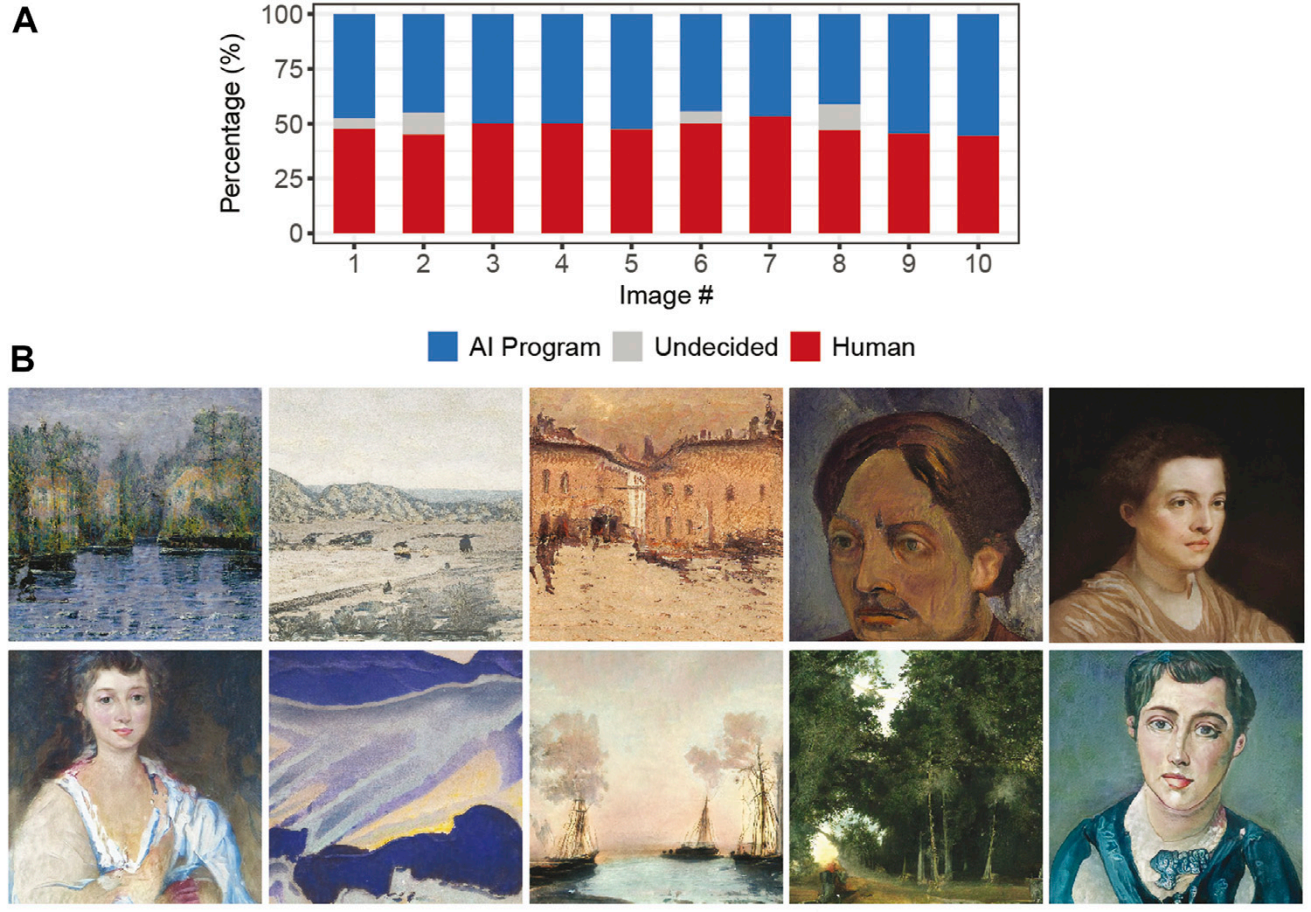
Distribution of respondents’ judgments of the top-10 images selected in our preliminary study for Studies 1 and 2 **(A)**. Selected images used in Studies 1 and 2 **(B)**.

None of the images had a majority of respondents being unsure about its provenance. We thus used Shannon Entropy to compute image ambiguity across responses indicating that humans or AI systems created the images. We selected the top-10 images in terms of ambiguity and used them for all subsequent studies. Of the ten images, five are landscapes, four are portraits, and one is an abstraction. Qualitative analysis of all 58 images showed that more realistic images were often perceived as human-created. On the other hand, abstractions were more frequently viewed as AI-generated. Figure 1A presents the distribution of responses for the selected images, and Figure 1B shows them. All images are made available in the study’s online repository for future research.

## 5 Study 1

Study 1 examined whether Gunkel’s social-relational approach to electronic agents’ moral standing could be applied to the context of AI-generative art. Our study employed a between-subjects design where participants interacted with AI-generated images either *before* or *after* evaluating the AI system’s moral status. Our analysis controlled for previous experiences with AI-generated images and treated the difference between participants in distinct treatment groups as the effect of participants’ interaction with the images in the system’s perceived moral status.

### 5.1 Methods

After consenting to the research terms, participants were told that some AI systems are currently being used to generate images and that they would be shown a series of them created by a specific model. Each participant was randomly assigned to one of two conditions. Participants assigned to the *pre* condition first responded to a series of questions compiled from previous work on mind perception theory (Gray et al., 2007; Bigman and Gray, 2018). Participants rated an AI system that can generate images concerning their perceived agency (e.g., to what extent the AI system “is intelligent;” six questions in total, see Supplementary Table S1) and experience (e.g., “can experience happiness;” six questions in total). We additionally asked participants to evaluate the system’s ability to create art (hereafter art agency) and experience art (hereafter art experience). All judgments were made on a 5-point scale from 0 (Not at all) to 4 (Extremely). Afterward, study participants were presented to all ten images selected in our Experimental Setting in random order. Participants were asked to evaluate each of the paintings in the range between $0 and $10,000.

Study participants assigned to the *post* condition responded to the same set of questions and art evaluations; however, in the opposite order, i.e., they first evaluated all ten images and then attended to the mind perception questionnaire. Participants did not differ in how long they spent evaluating the images (*t* (129.2) = 0.713, *p* = 0.48, *d* = 0.12) and rating the AI systems’ moral status (*t* (120.2) = −1.351, *p* = 0.18, *d* = 0.23) across conditions. All participants answered a series of demographic questions at the end of the study, including whether they had received any training in computer science or art-related subjects. We also gathered responses to a modified questionnaire of NARS (Negative Attitude towards Robot Scale) (Syrdal et al., 2009), with a modified text that covered “artificial intelligence programs” instead of “robots.”

### 5.2 Participants

Power analysis indicated that 128 participants were required for detecting a medium effect size (*η* ^2^ = 0.06) with the power of 0.80 and *α* = 0.05 (Campbell and Thompson, 2012). Hence, we recruited 160 respondents through the Prolific crowdsourcing platform. After removing respondents that failed an instructed response attention check question and those who had previously participated in a study where they had to evaluate AI-generated art (i.e., had interacted with AI-generated images before), our sample consisted of 140 participants (60 women, 77 men, three others) aged between 19 and 77 years old (mean = 31.96, SD = 11.96). We enforced the same recruitment conditions and payment as in Study 1.

### 5.3 Results

A principal component analysis (PCA) of participants’ attribution of moral status revealed two dimensions with eigenvalues larger than one (see Supplementary Tables S1 and S2). After varimax rotation, the first component (termed “experience”) accounted for all experience-related questions from the mind perception questionnaire with loadings greater than 0.78. The second factor (termed “agency”) included all agency-related questions with loadings greater than 0.65. We thus calculated a mean attribution of experience (Cronbach’s *α* = 0.93) and agency (*α* = 0.83) to the AI-generative system for each participant. Neither of the two principal components significantly accounted for art agency and experience (i.e., loadings were smaller than 0.6). These two variables were also not strongly correlated (*r* = 0.404, *p* < 0.001); we thus consider these two questions as distinct variables in our analysis.

The participants attributed moderate levels of agency (*M* = 1.85, *SD* = 0.96) and art agency (*M* = 2.59, *SD* = 1.16) to the AI-generative system. On the other hand, AI systems were rated as slightly able to experience art (*M* = 1.10, *SD* = 1.2) and were attributed almost no experience (*M* = 0.34, *SD* = 0.67). To what extent the study participants attributed agency (*M*_*pre*_ = 1.97, *M*_*post*_ = 1.74, *t* (136.5) = −1.427, *p* = 0.15, *d* = 0.24) and patiency (*M*_*pre*_ = 0.25, *M*_*post*_ = 0.42, *t* (132.9) = 1.531, *p* = 0.13, *d* = 0.25) to the AI system did not differ significantly across treatment conditions. Nevertheless, the participants attributed marginally higher levels of art agency (*M*_*pre*_ = 2.38, *M*_*post*_ = 2.77, *t* (125.6) = 1.981, *p* = 0.05, *d* = 0.34) and art experience (*M*_*pre*_ = 0.88, *M*_*post*_ = 1.29, *t* (135.1) = 2.119, *p* = 0.04, *d* = 0.35) to the generative model had they rated the system’s moral status after interacting with the images.

**FIGURE 2:**
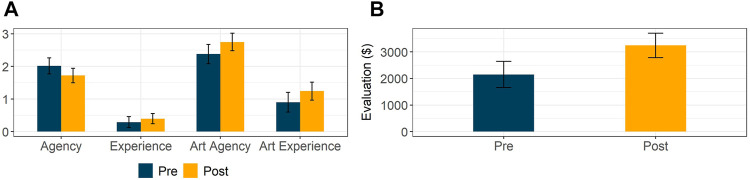
Attribution of agency, experience, art agency, and art experience to an AI system before and after being exposed to AI-generated paintings in Study 1 **(A)**. Marginal mean evaluation across all ten images depending on treatment group **(B)**.

The observations above raise the question of whether moral patiency and agency attribution differs across participants with distinct perceptions of AI-generated art, i.e., how each participant individually valued the presented images. We hence conducted an analysis of variance (ANOVA) accounting for the interaction between the study condition and the average value assigned to all images by each participant. We did not observe any significant effect of the treatment condition and its interaction with art evaluation across all dependent variables (*p* > 0.05 for all F-tests). We found the same results when controlling for respondents’ attitudes towards AI and their previous knowledge of computer science and art-related subjects. We present the estimated marginal means of all dependent variables and their corresponding 95% confidence intervals in Figure 2A.

An exploratory analysis of how participants evaluated the set of AI-generated paintings showed a large difference between respondents in distinct groups; those evaluating the images before attending to the mind perception questionnaire perceived the images to be more valuable (*M*_*pre*_ = 2,149, *M*_*post*_ = 3,244, *t* (137.9) = 3.244 *p* = 0.001, *d* = 0.55). A mixed-effects model regressing participants’ evaluation of all AI-generated paintings with treatment condition and image number as fixed effects indicated that respondents differed across conditions (*F* (1, 138) = 10.352, *p* = 0.002). We estimated marginal means across all ten images and found participants who evaluated all paintings before attending to the mind perception questionnaire to value them more highly (95% CI, *M*_*pre*_ = [1,657, 2,642], *M*_*post*_ = [2,786, 3,703], *p* = 0.002; see Figure 2B). We observed qualitatively similar results when accounting for respondents’ attitudes towards AI and their previous knowledge of computer science and art-related subjects.

### 5.4 Discussion

Whether participants interacted with AI-generated images before or after attributing moral agency and patiency to the system did not influence its perceived moral standing. We observed a significant difference in participants’ perception of the AI system’s capacity to create and experience art depending on the treatment condition. This effect, however, disappeared once we controlled for participants’ attitudes towards the AI systems’ outputs, i.e., the average price assigned to AI-generated art. It may well be the case that our proposed interaction with AI-generated art is not as strong a stimuli as the significant social interactions that authors defend to be crucial components of moral standing.

Nevertheless, study participants ascribed the ability to create art to the AI system although it was not described as an “artist,” nor their outputs were introduced as “art.” This specific artistic notion of the agency was perceived as more significant to the AI-generative system than the more general conception of agency captured by the mind perception questionnaire. In a similar vein, our results indicate that AI systems were attributed some ability to experience art even though they were not perceived to have the experience dimension of mind.

Finally, we observed a significant difference across treatment groups by expanding our analysis to how participants responded to AI-generated paintings. Even after controlling for individual variations through a mixed-effects model, AI-generated images were valued lower by participants who attributed moral standing to the AI system before interacting with its images. This result suggests that nudging participants to think about an AI system’s mind (e.g., its agency and patiency) could negatively influence how much they value its outputs. That is, the act of evaluating an AI system’s moral status could influence how people interact with them.

## 6 Study 2

Study 2 inquired whether Coeckelbergh’s socio-relational approach to electronic agents’ indirect moral status could be extended to the context of AI-generative art. The author suggests that electronic agents could be granted moral standing if others have a valuable relationship with them, i.e., one should respect these systems’ interests due to their extrinsic value. Hence, our study was designed to randomly assign participants to treatment groups that show how others perceived AI-generated images, e.g., by under- or overvaluing them.

### 6.1 Methods

After agreeing to the research terms, participants were told that some existing AI systems could generate images and that they would be shown some examples throughout the study. Each participant was randomly assigned to one of four treatment groups. Those assigned to the *pre* condition took part in a study similar to the *pre* condition in Study 1, i.e., they attributed moral status before interacting with a series of AI-generated images. Participants allocated to the *undervalue*, *median*, and *overvalue* conditions were presented a study design similar to Study 1’s *post* condition, where participants first evaluated a set of AI-generated paintings and then answered questions concerning their creator’s moral status.

Study 2 differed from the previous study in that participants were shown additional information during the art evaluation step. After evaluating each of the images, participants were shown how other respondents evaluated the same painting depending on the treatment condition they were assigned to. They were subsequently asked to modify their initial evaluation if they desired to do so. Participants assigned to *pre* and *median* conditions were shown median values calculated from Study 1’s responses.^1^ Those in the *undervalue* and *overvalue* groups were presented to evaluations three times lower or larger than those presented in the other two conditions. This design choice aimed to elucidate the AI system’s extrinsic value, which Coeckelbergh argues to be crucial for electronic agents’ moral standing.

All participants responded to the same mind perception questionnaire and art-related questions from Study 1. We additionally asked participants to rate the AI-generative system’s moral standing concerning six statements. Respondents were asked to what extent the system “has legitimate interests,” “can have rights,” “has inherent value,” “is more than just a tool,” “deserves protection,” and “deserves moral consideration.” These questions were created after an extensive review of the recent literature addressing the moral standing of electronic agents (Gunkel, 2018a; Coeckelbergh, 2020b; Gordon, 2020). All judgments were made on a 5-point scale from 0 (Not at all) to 4 (Extremely). Participants did not differ in how long they spent evaluating the images (all *p* > 0.05 after Bonferroni corrections) and rating the AI systems’ moral status (all *p* > 0.05) across conditions. Finally, participants were asked the same demographic and personal experience questions from Study 1 before completing the study.

### 6.2 Participants

Considering the power analysis conducted for Study 1, we decided to double the number of participants recruited for this study to account for doubled treatment conditions. We thus recruited 315 respondents through Prolific. After removing respondents that failed an attention check question similar to Study 1’s and those who had previously participated in a study where they had to evaluate AI-generated art, our sample consisted of 263 participants (126 women, 134 men, three others) aged between 19 and 75 years old (mean = 34.40, SD = 12.73). Recruitment requirements and conditions were the same as in previous studies.

### 6.3 Results

We identified four principal components with eigenvalues larger than one by analyzing participants’ ratings of the AI system’s moral status (see Supplementary Tables S3 and S4). The first two components accounted for all of the experience- and agency-related questions with loadings greater than 0.84 and 0.69, respectively. In a similar manner to Study 1, we calculated mean attributions of experience (*α* = 0.96) and agency (*α* = 0.88) for each participant. The third factor identified by the principal component analysis included five out of the six novel moral standing-related questions (with loadings greater than 0.61). In contrast, the last factor accounted for this extra item (“has inherent value,” loading equal to 0.69) and art agency (loading equal to 0.87). We again kept art agency and experience as independent variables due to their low correlation (*r* = 0.411, *p* < 0.0001). We finally calculated participants’ mean attribution of moral status by averaging all items proposed by this study (*α* = 0.86). All results discussed below are qualitatively similar to those controlling for participants’ attitudes towards AI and their previous knowledge of computer science and art-related subjects.

**FIGURE 3:**
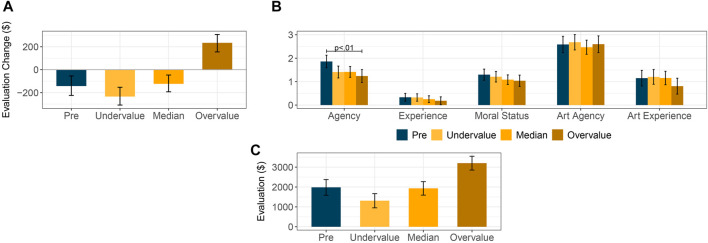
To what extent participants modified their initial art evaluation after treatment in Study 2 **(A)**. Attributions of agency, experience, moral status, art agency, and art experience to the AI system depending on the condition participants were assigned to in Study 2 **(B)**. Marginal mean evaluation across all ten images depending on treatment group **(C)**.

As a manipulation check, we analyzed whether treatment groups differed in how much participants modified their initial evaluation after seeing others’ judgments. We ran a mixed-effects model regressing evaluation-change with the study condition and the image number as fixed effects. Participants’ initial evaluation was included as a covariate. The results suggest that the condition to which participants were assigned played a role in how much they changed their initial evaluation (*F* (3, 220) = 26.684, *p* < 0.001). Pairwise comparisons between marginal means across all images show that participants presented to overvalued AI-generated art increased their initial evaluation after treatment. In contrast, those assigned to all others conditions decreased their evaluation—we note that evaluation-change did not significantly differ between the *pre*, *median*, and *undervalue* conditions (see Figure 3A).

Similarly to Study 1, participants attributed moderate levels of agency (*M* = 1.45, *SD* = 1.00) and art agency (*M* = 2.54, *SD* = 1.27) to the AI system, while it was rated as slightly capable of experiencing art (*M* = 1.09, *SD* = 1.27). Participants attributed low levels of experience (*M* = 0.28, *SD* = 0.63) and moral status (*M* = 1.17, *SD* = 0.90) to the automated system. Pairwise t-tests between study conditions only suggested a significant difference in the attribution of agency. After Bonferroni corrections, we observed that participants presented overvalued AI art attributed lower levels of agency to their creator than those who evaluated it before interacting with the AI-generated images (*M*_*pre*_ = 1.86, *M*_*overvalue*_ = 1.31, *t* (1,102) = −3.02, *p* = 0.02, *d* = 0.55; all others *p* > 0.05).

Having found non-significant differences in evaluation-change across treatments, we analyzed ANOVA models with study conditions and their interaction with the extent to which participants changed their initial evaluation (i.e., the treatment effect) as fixed effects. Respondents’ average initial art evaluation was included as a covariate. There were significant differences across treatment groups for the AI system’s perceived agency (*F* (3, 254) = 3.985. *p* < 0.01). The estimated marginal means showed higher attributions of agency by participants in the *pre* condition vis-as-vis those in the *overvalue* treatment group (95% CI, *M*_*pre*_ = [1.59, 2.13], *M*_*overvalue*_ = [0.97, 1.51], *p* = 0.01; see Figure 3B). To what extent participants attributed all other variables did not differ across conditions (*p* > 0.05 for all F-tests).

Finally, we analyzed how differently participants evaluated the AI-generated paintings they were shown depending on the study condition they were assigned to. We ran a mixed-effects model with the experimental condition and image number as fixed effects and evaluation-change as a covariate. We included the interaction term between the study condition and the evaluation change to account for the non-significant contrasts between some treatment conditions. Here, the condition played a significant role in how participants evaluated the AI-generated images (*F* (3, 259) = 20.235, *p* < 0.001). As expected from the treatment condition, participants assigned to the *overvalue* condition evaluated AI-generated images more highly in comparison to those in all other conditions (95% CI, *M*_*pre*_ = [1,586, 2,375], *M*_*undervalue*_ = [953, 1,667], *M*_*median*_ = [1,593, 2,271], *M*_*overvalue*_ = [2,852, 3,547], all *p* < 0.001,; see Figure 3C). All other contrasts were not significant (*p* > 0.05).

### 6.4 Discussion

Similarly to Study 1, participants attributed higher levels of art-related agency and experience than their more general (and moral) counterparts to the AI-generative system. The result was again observed without explicitly introducing the AI system as an “artist” or its outputs as “art.” Our results reveal that participants attributed experience, moral status, art agency, and art experience regardless of our study’s nudges concerning the AI-generative model’s extrinsic value. In contrast, participants showed a distinction concerning the AI system’s perceived agency—overvaluing the system’s outputs led to a lower perceived agency in comparison to ratings prior to interacting with AI-generated art.

We expanded Study 2 to include a novel measure of perceived moral standing independent of an entity’s perceived experience covered by the mind perception questionnaire. This was done because the social-relational approach to electronic agents’ moral standing challenges perspectives that defend experience-related capacities as preconditions for moral status. Nevertheless, we did not find any significant difference between treatment conditions in both attributions of experience and our proposed moral standing measure. These results corroborate our findings from Study 1 by showing that interacting with AI-generated outputs should not influence people’s ascription of moral standing.

Nudging people to think about the mind of an AI system did not necessarily influence how they valued AI-generated art in Study 2. Our results instead suggest that overvaluing AI-generated art could influence how people perceive it. We hypothesize that the treatment conditions’ social influence mitigated any possible effect of considerations about an AI system’s mind similar to those found in Study 1. Similar to how past auctions of AI-generated art were presented to the public (Cohn, 2018; Ives, 2021), overvaluing these outputs could influence how much people value them.

## 7 General Discussion

Inspired by Gunkel’s and Coeckelbergh’s social-relational approaches to robots’ moral standing, we conducted two studies to understand whether a similar perspective would influence people’s ascription of moral status to a nonsocial automated agent, namely an AI-generative system. We first identified a set of ten AI-generated images that were used in subsequent studies. Study 1 inquired whether interacting with these images would influence people’s ascription of moral agency and patiency to their creator—as suggested by Gunkel (2018b). Study 2 asked whether highlighting an AI system’s extrinsic value by undervaluing or overvaluing its images affected participants’ attribution of agency, experience, and moral status, as proposed by Coeckelbergh (2020b). The current research took a novel experimental approach to the normative debate of robot rights in the context of AI-generated art.

We employed a series of measures to quantify AI systems’ perceived moral (and artistic) standing. Interacting with AI-generated art did not significantly impact how participants perceived the system’s ability to create art, experience art, and the experience dimension of mind in both Studies 1 and 2. The latter was measured by a mind perception questionnaire, whose measure has been shown to correlate with the recognition of moral rights (Waytz et al., 2010; Gray et al., 2007). Study 2 also showed that interacting with AI-generated art did not influence the AI system’s perceived moral standing in a novel measure of moral consideration independent of the system’s experience.

Study 2’s participants attributed lower levels of agency to AI systems after interacting with overvalued AI-generated art. This finding suggests that seeing others overvaluing AI systems’ abilities could negatively influence their perceived agency. This finding may be contrary to what one would expect. Similar to Coeckelbergh’s approach to AI systems’ patiency, highlighting the system’s creative value by overvaluing its generated images should, at first thought, increase their perceived (artistic) agency.

Finally, Study 1 suggests that nudging participants to think about an AI systems’ mind could lead to a lower appreciation of AI-generated art. A possible interpretation is that machine creativity is not valued to the same extent as its human counterparts, particularly when AI systems’ lack of humanness and mind becomes apparent. As argued by some scholars, AI-generated art may lack the meaning necessary to be considered art—such meaning can only emerge from human artistic communication (Elgammal, 2020). Another possible explanation is that art is also evaluated by the effort put into its creation. More realistic images in our Experimental Setting were often attributed to human artists, while abstractions were usually viewed as AI-generated. Participants might have judged the generation process of an AI-generated art not as labor and particularly mind intensive as human-created art. As one participant has put it in an open-ended comment to our study, “knowing that an AI made it devalues [the image].”

### 7.1 Limitations and Future Work

Both studies have found AI-generative systems being perceived as an agent and patient to a higher level for their particular artistic abilities. Under the social paradigm of art described above, participants included AI systems in their art world. Most AI systems are proficient in a narrow task, such as generating images, and our results suggest that participants rate their agency and patiency similarly. This observation raises the question of how participants would ascribe moral status to an AI system that is explicitly described as a moral agent or patient. For instance, scholars have proposed the creation of “artificial moral agents” capable of identifying and resolving moral dilemmas (Wallach and Allen, 2008). Past research has also explored how people interact with robots described as emotional (de Melo et al., 2014; Lee et al., 2021). A future line of research could inquire how social interactions with AI systems with different abilities would affect their perceived moral standing.

Presenting participants with others’ judgments of an AI system’s outputs, as done in Study 2, seems to influence their evaluation negatively. Although this effect was countered by others’ overvaluation of AI-generative art, which led participants to increase their initial evaluation, respondents appear to decrease their initial evaluation even if presented with other participants’ median judgments. As shown by Study 1, making participants think about the AI-generative system’s lack of mind decreased how much they value its outputs. Similarly, forcing participants to think more about AI-generated art influenced how much they value it. Future work may study how nudging people to think (harder) about an AI system’s (lack of) mind and its outputs may influence how participants evaluate its creations.

The current research examined a growing research area, namely AI-generative models. Extensive research has been devoted to developing and improving generative systems (e.g., Ramesh et al. (2021); Brown et al. (2020)), and many of them are already deployed in the wild (Warren, 2020; Dorrier, 2021). Our results, however, may not extend to other applications of AI systems. For instance, in the context of social robots, Darling (2016) has presented a series of anecdotes suggesting that people desire to protect social robots after interacting with them. Future research in a wide range of applications is needed to explore how people might perceive AI systems’ and robots’ moral standing in different environments.

We have explored Gunkel’s and Coeckelbergh’s social-relational perspective on robots’ moral standing in the context of AI-generated art. This setting was chosen for its prominence in the AI research agenda, its legal and moral issues (e.g., concerning copyright law), and the widespread attention to AI-generated art auctions worldwide. Although art does contain a social dimension, our studies’ stimuli may not have simulated the social interactions proposed by both authors in their theses. Nevertheless, we empirically explored both perspectives in a setting that was yet to be comprehensively investigated by previous experimental and normative research.

Our results confront the thesis that property-based grounds for moral patiency can be entirely substituted by social-relational perspectives (Coeckelbergh, 2010) in that considerations about the mind of non-humans, i.e., a form of ontological consideration, may influence future interactions. This finding suggests that even if social-relational approaches can ground the moral standing of machines, they may not be entirely detached from the property-based views they challenge. Instead, the property and relational approaches can be intertwined in justifying moral standing, as discussed by Gellers (2020).

Our findings contribute extensively to the discussion concerning AI systems’ and robots’ moral status. Our results provide scholars with empirical evidence and methods that can influence future normative discussion on the topic. For instance, we found that nudging participants to think about AI systems’ (lack of) mind could influence future social interactions in the context of AI-generated art, which is an important addition to the social-relational perspectives studied in this paper. We call for future research that empirically examines normative debates on AI systems’ and robots’ moral agency and patiency so that subsequent discussions concerning how automated agents should be included in our moral and social spheres can make fruitful progress.

## Data Availability

The datasets and scripts used for analysis presented in this study can be found at https://github.com/thegcamilo/AIArt_MoralStanding.

## Footnotes

1 Due to a programming error, median values were calculated with respect to the order images were shown to participants in Study 1. For instance, image #1’s median value was determined by the median evaluation of the first image shown to each participant. Note that the image order was randomized between participants. Our study conditions should not be affected by this error, i.e., all images were overvalued or undervalued on their respective treatment conditions.
