# Supplementary material for: On the Social-Relational Moral Standing of AI: An Empirical Study Using AI-Generated Art
Source: Front Robot AI. 2021 Aug 5;8:719944. doi: 10.3389/frobt.2021.719944 (PMC8375468; doi:10.3389/frobt.2021.719944)
Supplement: Supplementary file 1 [file DataSheet1.pdf]

frontiers

Supplementary Material

1 SUPPLEMENTARY TABLES

	RC1	RC2	To what extent do you think the AI program...
Agency Q1	0.179	**0.648**	Can communicate with others
Agency Q2	0.386	**0.689**	Is able of thinking
Agency Q3	0.125	**0.651**	Plans his actions
Agency Q4	0.018	**0.824**	Is intelligent
Agency Q5	0.192	**0.617**	Has foresight
Agency Q6	0.164	**0.786**	Is able to think things through
Experience Q1	**0.819**	0.120	Is sensitive to pain
Experience Q2	**0.797**	0.241	Can experience happiness
Experience Q3	**0.894**	0.109	Can experience fear
Experience Q4	**0.883**	0.213	Can experience compassion
Experience Q5	**0.783**	0.285	Can experience empathy
Experience Q6	**0.881**	0.090	Can experience guilt
Art Agency	0.097	0.554	Can create art
Art Experience	0.528	0.487	Can experience art
Eigenvalues	6.28	2.30	
Explained Variance	0.34	0.27	
Subscale Cronbach's α	0.93	0.83	

Table S1. Loading matrix of principal component analysis (PCA) after varimax rotation on participants' attributions of agency, experience, art agency, art experience in Study 1.

	Agency	Experience	Art Agency	Art Experience
Agency	1	0.439	0.410	0.511
Experience	0.439	1	0.221	0.568
Art Agency	0.410	0.221	1	0.404
Art Experience	0.511	0.568	0.404	1

Table S2. Pairwise Pearson correlation (r) between variables covered by Study 1.

	RC1	RC2	RC3	RC4
Agency Q1	0.102	**0.692**	0.256	−0.002
Agency Q2	0.264	**0.787**	0.193	0.121
Agency Q3	0.146	**0.760**	0.121	0.066
Agency Q4	0.054	**0.731**	0.152	0.308
Agency Q5	0.256	**0.702**	0.196	0.029
Agency Q6	0.221	**0.813**	0.098	0.143
Experience Q1	**0.861**	0.102	0.116	0.074
Experience Q2	**0.840**	0.240	0.242	0.120
Experience Q3	**0.891**	0.144	0.217	0.027
Experience Q4	**0.860**	0.227	0.231	0.105
Experience Q5	**0.901**	0.204	0.194	0.093
Experience Q6	**0.859**	0.179	0.136	0.033
Moral Status Q1	0.446	0.361	**0.606**	0.068
Moral Status Q2	0.518	0.261	**0.605**	0.061
Moral Status Q3	0.087	0.054	**0.429**	0.690
Moral Status Q4	0.159	0.203	**0.747**	0.169
Moral Status Q5	0.127	0.209	**0.738**	0.325
Moral Status Q6	0.334	0.210	**0.735**	0.020
Art Agency	0.063	0.223	0.028	0.866
Art Experience	0.480	0.226	0.286	0.488
Eigenvalues	9.26	2.51	1.61	1.09
Explained Variance	0.28	0.20	0.15	0.09
Subscale Cronbach's α	0.96	0.88	0.86	—

Table S3. Loading matrix of principal component analysis (PCA) after varimax rotation on participants' attributions of agency, experience, moral status, art agency, art experience in Study 2. Questions are presented in Table S1 and Section 6 in the main text.

	Agency	Experience	Art Agency	Art Experience	Moral Status
Agency	1	0.455	0.341	0.440	0.555
Experience	0.455	1	0.184	0.574	0.588
Art Agency	0.341	0.184	1	0.411	0.363
Art Experience	0.440	0.574	0.411	1	0.581
Moral Status	0.555	0.588	0.363	0.581	1

Table S4. Pairwise Pearson correlation (r) between variables covered by Study 2.

2 SUPPLEMENTARY FIGURES

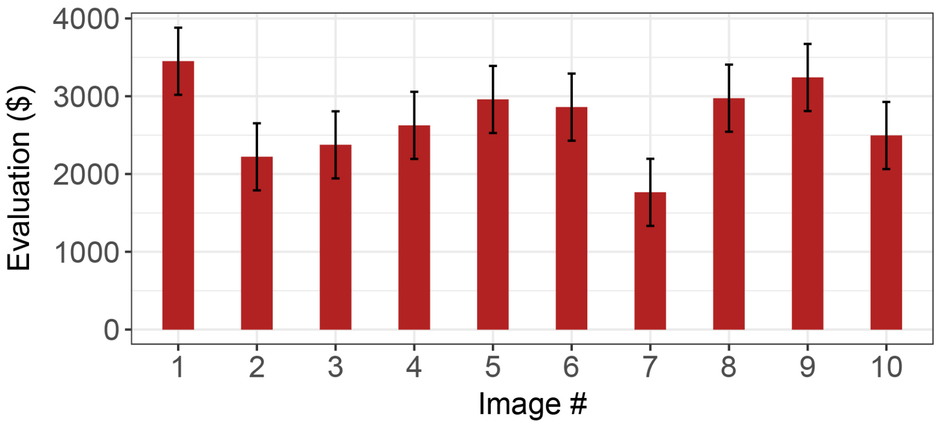

Figure S1. Marginal mean image evaluation in Study 1.

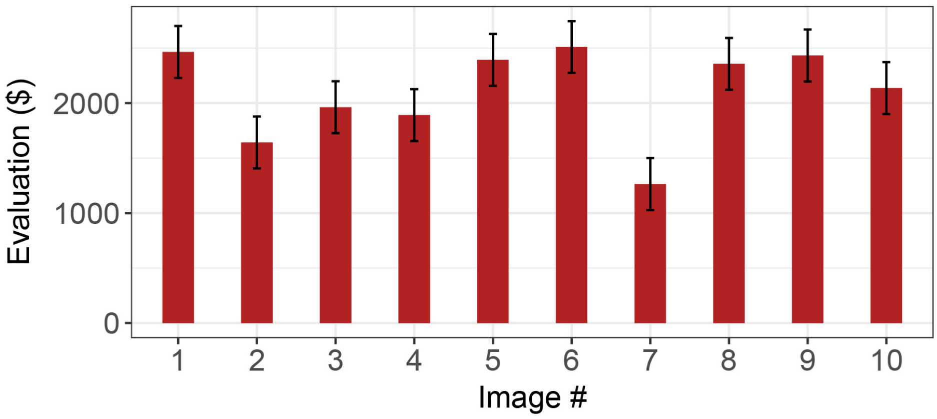

Figure S2. Marginal mean image evaluation in Study 2.

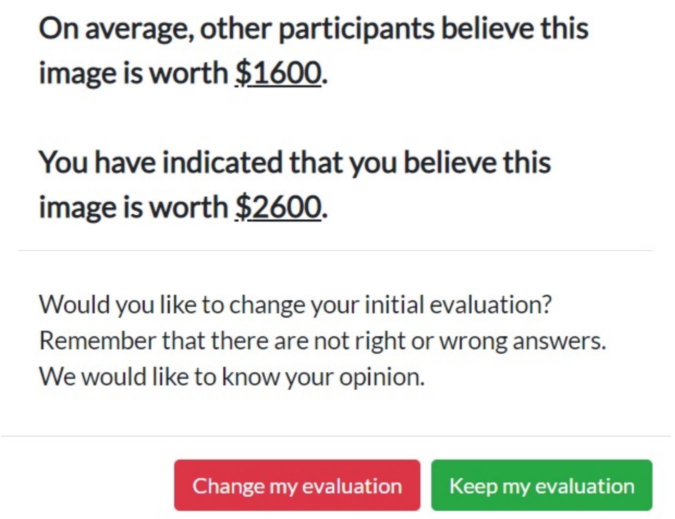

Figure S3. Example pop-up shown to participants in Study 2 asking whether they would like to change their initial evaluation.
